# Impact of genetic variant of *HIPK2* on the risk of severe radiation pneumonitis in lung cancer patients treated with radiation therapy

**DOI:** 10.1186/s13014-019-1456-0

**Published:** 2020-01-08

**Authors:** Yang Tang, Li Yang, Wan Qin, Min’ Xiao Yi, Bo Liu, Xiang’Lin Yuan

**Affiliations:** 1 Department of Oncology, Tongji Hospital, Huazhong University of Science and Technology, Wuhan, Hubei Province China; 2 Department of Hematology, Tongji Hospital, Huazhong University of Science and Technology, Wuhan, Hubei Province China

**Keywords:** Radiation pneumonitis, Lung cancer, *HIPK2*, SNP

## Abstract

**Background:**

Homeodomain-interacting protein kinase 2 (*HIPK2*) has increasingly drawn attention as recent researches demonstrated its unique role in the regulation of multiple fundamental processes such as apoptosis, proliferation and DNA damage repair. Most importantly, *HIPK2* was shown to play regulatory role in inflammation and influence the phenotype and activity of fibroblasts. In this study, we aimed to evaluate the impact of *HIPK2* gene variant on risk of radiation pneumonitis for patients with pulmonary malignancies.

**Methods:**

169 lung cancer patients with radiotherapy were included in our prospective study and genotyped by Sanger Sequence method. Multivariable Cox hazard analysis and multiple testing were applied to estimate the hazard ratio (HR) and 95% confidence intervals (CIs) of all factors possibly related to the risk of radiation pneumonitis (RP).

**Results:**

Patients with Mean Lung Dose (MLD) ≥ 15Gy, Lung V_20_ ≥ 24% had higher risk of RP ≥ grade 2 compared with those counterparts (HR = 1.888, 95% CI: 1.186–3.004, *P* = 0.007; HR = 2.126, 95% CI: 1.338–3.378, *P* = 0.001, respectively). Importantly, CC genotype of *HIPK2:* rs2030712 were strongly related to an increased occurrence of RP ≥ grade 2 (HR = 2.146, 95% CI: 1.215–3.791, *P* = 0.009).

**Conclusion:**

*HIPK2:* rs2030712 was found to be significantly related to RP of grade ≥ 2 in our cohort, and may thus be one of the important predictors of severe RP before radiotherapy, if further validated in larger population.

**Trial registration:**

Our study was prospective and observational. The research was registered in ClinicalTrials.gov database as NCT02490319.

## Background

Globally, lung cancer currently remains as the top one cause of cancer-related mortality. According to the latest statistical report, there are 2.1 million new lung cancer cases and 1.8 million deaths predicted globally in 2018, nearly close to 1 in 5 (18.4%) of all cancer deaths [1]. Among all countries worldwide, China suffered from high rates of male lung cancer (above 40 per 100,000) in 2018. Radiotherapy (RT), with or without chemotherapy, still acts as the fundamental treatment for lung cancer patients. However, the efficacy of radiotherapy is restrained due to a series of RT-related complications that cause patients intolerance.

Radiation pneumonitis (RP) is a type of inflammation and subsequent fibrosis that occurs after irradiation, which is the most common complication and the major dose-limiting toxicity associated with radiotherapy. By limiting the radiation dose that can be applied and the size of the irradiated volume, RP hinders the tumor-controlling effects of radiotherapy [2, 3]. Furthermore, poor quality of life or life-threatening symptoms can be caused by RP in 15–40% of all patients who are irradiated for lung cancer [4]. Therefore, reliable predictors for RP occurrence is of great value to maximize the therapeutic effects and to minimize its adverse effects of RT. In addition to the previous reported patient- and treatment-related factors [5], including Karnofsky performance status (KPS), chronic lung disease [6], smoking status, chemotherapy [7, 8], dosimetric parameters and plasma values of TGFβ [9, 10], some genetic variants were recently found to be associated with the occurrence and development of RP [11–16].

Homeodomain-interacting protein kinase 2 (*HIPK2*) is a member of serine/threonine kinase family. *HIPK2* plays important part in phosphorylation and interaction with a series of molecules involving development gene transcription and cellular responses to stress signals [17]. In addition, *HIPK2* regulates multiple transcription factors functioning in different processes including differentiation, apoptosis and proliferation [18]. Recently, the function of *HIPK2* in regulation of inflammation and fibrosis has drawn much attention. A study of idiopathic lung fibrosis (IPF) patients indicated that *HIPK2* gene defect was found in fibroblastic foci and such defect may result in phenotypic and biological behavioral change of fibroblasts and myofibroblasts [19]. The results provided evidences that dysfunction of *HIPK2* play significant role in disease progression and treatment resistance for IPF patients. However, up till now, the research on the function of *HIPK2* in RP risk and pathogenesis is lacking. HIPK2 rs2030712 has been investigated as a potential risk factor of chronic kidney fibrotic disease occurrence and progression. Despite the fact that the study presented negative result [20], considering race disparities and different disease pathogenesis of RP, we selected rs2030712 as single nucleotide polymorphism (SNP) candidate for this study. In order to identify clinical valuable SNPs on RP occurrence and severity, in this study we explored the association between *HIPK2* SNP rs2030712 with RP risk in our cohort.

## Methods

### Patient population

Our prospective study was registered in ClinicalTrials.gov database (NCT02490319). In brief, 190 lung cancer patients were initially enrolled. All patients were treated with radiation therapy at Tongji Hospital, Huazhong University of Science and Technology (Wuhan, Hubei Province, China) between 2009 and 2015. We included the patients with a radiation dose at least 45 Gy, age > 18 years old, KPS > 60 and a life expectancy of at least 6 months. Patients with previous thoracic irradiation or severe cardiopulmonary diseases were excluded from our study. Of the 199 patients, 169 patients (114 with non-small cell lung cancer and 55 with small-cell lung cancer) were eventually included for the final genotyping analysis. Samples from 169 patients were genotyped by Sanger Sequencing for the SNP candidate. This study was approved by the Review Board of Tongji Hospital. Written informed consents were obtained from all patients for the use of their clinical information and for obtaining their blood and DNA.

### Treatment and follow-up

All patients received radiotherapy with 6-MV X-rays from a linear accelerator (Elekta Synergy, Elekta, Sweden). The median total radiation dose was 56 Gy (range from 45 to 66 Gy), with 1.5 to 2 Gy administered per radiation treatment. IMRT (intensity-modulated radiation therapy) was administered to 46.7% of patients (*n* = 79). Computed tomography simulation (CT/e, GE, Fairfield, Connecticut, USA) was performed before the RT treatment was planned. The target volumes and critical normal organs were delineated by the three-dimensional planning system (Pinnacle Version 9.2). The baseline clinical characteristics and treatment details of the patients are shown in Table 1.

**Table 1:** Detailed clinical characteristics of the patients enrolled in this study (*N* = 169)

Characteristic	No. of Patients	%
Sex		
Male	125	74.0
Female	44	26.0
Age, years		
Median	58	
Range	28–78	
Histology		
SCLC	55	32.5
NSCLC	114	67.5
Stage		
I- II	24	10.2
III-IV	145	85.8
KPS		
80–100	123	72.6
< 80	46	27.4
Smoking		
Smoker	106	62.0
Non-smoker	63	38.0
Chemotherapy		
Yes	160	94.7
No	9	5.3
CRT		
Yes	44	26.0
No	125	74.0
Surgery		
Yes	86	50.9
No	83	49.1
IMRT		
Yes	79	46.7
No	90	53.3
Radiation dose (cGy)		
Median	5600	
Range	4500–6600	
MLD (cGy)		
Median	1368	
Range	178–2017	
V_20_		
Median	24.82	
Range	0–42.00	
COPD		
Yes	19	11.2
No	150	88.8

Abbreviations: *KPS* Kamofsky performance status; *CRT* concurrent chemoradiation; *IMRT* intensity-modulated radiation therapy; *MLD* mean lung dose; *V*_*20*_ volume of normal lung receiving 20 Gy or more radiation; *COPD* chronic obstructive pulmonary disease

All patients enrolled in this study were examined during and one month after radiotherapy. Then, the patients were followed every three months for the first year and every six months thereafter. At each follow-up visits, all patients were asked to undergo a chest X-ray or CT and clinical information, including symptoms, was collected. RP was graded by two radiation oncologists (associate chief physician level required, with minimum 5 years of clinical experiences) according to the Common Terminology Criteria for Adverse Events 4.0 as follows: Grade 0, no change; Grade 1, asymptomatic and diagnosed by radiographic findings only; Grade 2, symptomatic, not interfering with daily activities; Grade 3, symptomatic, interfering with daily activities or oxygen required; Grade 4, assisted ventilation required; Grade 5, fatal.

### Genotyping methods

Genomic DNA was extracted with a PureLink Genomic DNA Mini Kit (Invitrogen, K1820–01) from peripheral blood. *HIPK2*: rs2030712 was selected as single SNP candidate in this study, and was genotyped by Sanger Sequencing method for samples from 169 patients included. The primer pairs for rs2030712 were F: 5′- TGGAGATTTACAACACTCTAGGG -3′; R: 5′- ACAGAACTCACGTGTGCTTT − 3′. The 262 bp PCR products were then subjected to DNA sequencing to detect mutations.

### Statistical analysis

The end point for this study was the development of RP ≥ grade 2. The time to the end point was calculated from the start of radiotherapy. Patients who did not experience RP ≥ grade 2 within 12 months of RT were censored. SPSS 21.0 statistical software (SPSS, lnc., Chicago, IL, USA) was used for the statistically analysis. Patients were divided into groups according to their genotypes, and Cox proportional hazard analysis was applied to estimate the hazard ratio (HR) and 95% confidence intervals (CIs) of all factors possibly related to the risk of RP. Moreover, multivariable Cox regression analysis was used for the adjustment of covariates. The influences of the genotypes on RP risk were assessed by Kaplan-Meier analysis and compared with log-rank tests.

## Results

### Patient characteristics and radiation pneumonitis

One hundred sixty-nine patients were included in this study with 125 males and 44 females. Their characteristics are listed in Table 1. The median age of the population was 58 years (range from 28 to 78 years); 114 patients had NSCLC, and 55 had SCLC. In the study cohort, 85.5% of patients had stage III-IV disease, 50.9% underwent surgery before RT, almost all patients (94.7%) received induction chemotherapy followed by radiotherapy and 26.0% had concurrent chemoradiation. The median radiation dose was 56 Gy (range from 45 to 66 Gy), the median mean lung dosage (MLD) was 13.68 Gy (range from 1.78 to 20.17 Gy), and the median V_20_ was 24% (range from 0 to 42.00%).

Within 12 months of radiotherapy, 99 patients (58.6%) suffered RP ≥ grade 2. The associations between patient-, tumor- and therapy-related characteristics and RP ≥ grade 2 are listed in Table 2. The univariable and multivariable analysis by Cox regression model revealed that MLD and V_20_ was significantly related to RP ≥ grade 2. Patients with elder age, MLD ≥ 15Gy, V_20_ ≥ 24% had higher risk of RP ≥ grade 2 compared with those counterparts (HR = 1.888, 95% CI: 1.186–3.004, *P* = 0.007; HR = 2.126, 95% CI: 1.338–3.378, *P* = 0.001, respectively) (Table 2), which were consistent with the results of other publications.

**Table 2:** Association between patient-, tumor-, and therapy-related characteristics and Grade ≥ 2 radiation pneumonitis (*N* = 169)

Parameter	Univariable Analysis			Multivariable Analysis		
	HR	95% CI	P	HR	95% CI	P
Sex						
Female	1			1		
Male	1.216	0.773–1.915	0.398	1.379	0.758–2.511	0.292
Age, years						
< 58	1			1		
≥ 58	1.413	0.951–2.098	0.087	1.541	0.997–2.383	0.052
Histology						
SCLC	1			1		
NSCLC	1.195	0.791–1.804	0.398	1.251	0.727–2.153	0.418
Stage						
I-II	1			1		
III-IV	1.100	0.601–2.013	0.758	1.132	0.593–2.163	0.707
KPS						
80–100	1			1		
< 80	1.341	0.877–2.052	0.176	1.566	0.993–2.470	0.054
Smoking						
Smoker	1			1		
Nonsmoker	0.926	0.619–1.386	0.708	0.964	0.337–2.192	0.435
Surgery						
Yes	1			1		
No	1.014	0.684–1.504	0.945	0.690	0.390–1.223	0.204
Chemotherapy						
Yes	1	1		1		
No	0.500	0.203–1.233	0.132	0.473	0.189–1.187	0.111
CRT						
Yes	1			1		
No	0.843	0.529–1.344	0.472	0.956	0.575–1.588	0.861
IMRT						
Yes	1			1		
No	1.077	0.726–1.598	0.712	1.098	0.710–1.699	0.675
Radiation dose, cGy						
<5600	1			1		
≥ 5600	1.083	0.729–1.610	0.692	1.139	0.633–2.535	0.737
MLD, cGy						
< 1500	1			1		
≥ 1500	1.510	1.093–2.235	0.045	1.888	1.186–3.004	**0.007**
V20						
< 24%	1			1		
≥ 24%	1.730	1.138–2.631	0.010	2.126	1.338–3.378	**0.001**
COPD						
Yes	1			1		
No	0.639	0.246–1.661	0.358	0.780	0.339–1.998	0.472

Note: Multivariable analyses were adjusted for all of the factors in this table, statistically significant *p* values for multivariable analysis were shown in boldface.

^*^Either MLD or V20 was used in the multivariable analyses, but not both

### *HIPK2* SNPs and RP

**Fig. 1:**
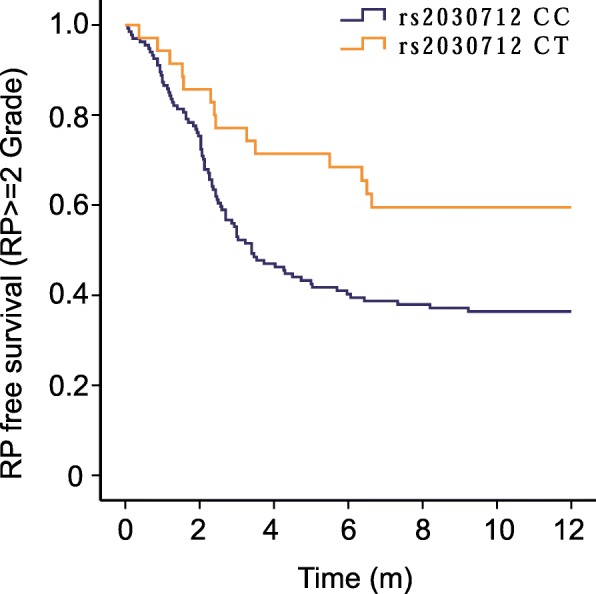
Kaplan-Meier estimates RP-free survival (RP ≥ grade 2) for each genotype of *HIPK2*:rs2030712

*HIPK2:* rs2030712 was found to be significantly associated with the occurrence of RP ≥ grade 2 (Table 3). Figure 1 is a plot of the RP-free survival percentage for RP ≥ grade 2 for each genotype of *HIPK2:* rs2030712 determined by the Kaplan-Meier method. Patients with the CC genotype of *HIPK2:* rs2030712 had significantly higher risks of RP ≥ grade 2 than patients with CT genotype (*P* < 0.0001). Furthermore, multiple Cox proportional hazard analyses with adjustments for all of the characteristics listed in Table 1 revealed that the CC genotype of *HIPK2:* rs2030712 were strongly related to a increased occurrence of RP ≥ grade 2 (HR = 2.146, 95% CI: 1.215–3.791, *P* = 0.009) (Table 3).

**Table 3:** Association between genotypes and Grade ≥ 2 RP

Polymorphism and Genotype	No.of event	No.of total	Univariable analysis			Multivariable analysis		
			HR	95% CL	P	HR	95% CL	P
*HIPK2*: rs2030712								
CT	14	35	1			1		
CC	85	134	2.009	1.140–3.539	0.016	2.146	1.215–3.791	0.009

NOTE: Multiple analyses in this table were adjusted for all the factors listed in Table 1

Abbreviations: *HR* hazard ratio; *CI* confidence interval

Pc: *P*-value corrected by Benjamini and Hochberg False Discovery Rate correction

### *HIPK2: rs2030712* and Dosimetric factors

**Fig. 2:**
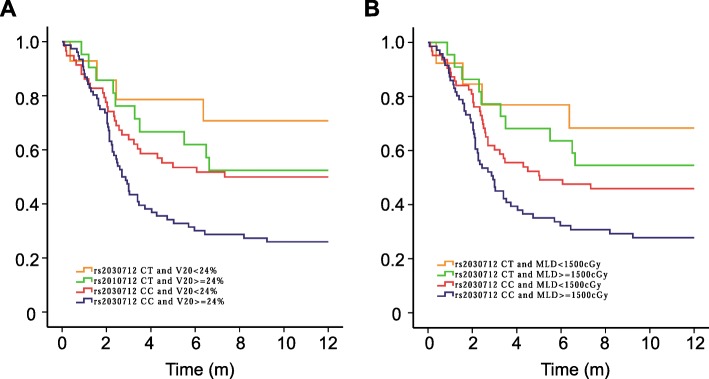
Kaplan-Meier estimates effect of genotype in *HIPK2*:rs2030712 and dosimetric parameters on RP-free survival (RP ≥ grade 3) (**a**) *HIPK2*:rs2030712 and MLD; (**b**) *HIPK2*:rs2030712 and V_20_

Patients were divided to four groups based on the dosimetric factors-V_20_ or MLD and *HIPK2:* rs2030712 genotypes in order to evaluate the impact of the *HIPK2:* rs2030712 genotypes on RP in different dosimetric groups. Patients with CC genotype of *HIPK2:* rs2030712 and MLD ≥ 15Gy or V20 ≥ 24% had the highest risk of RP grade ≥ 2 compared with other groups (*P* < 0.0001 and *P* < 0.0001, respectively, Fig. 2a, b). Interestingly, for the patients with *HIPK2:* rs2030712 CC genotype and MLD <15Gy or V20 < 24%, they had even higher incidence of RP ≥ grade 2 with the patients who received MLD more than 15Gy or V20 more than 24%, suggesting the dominant and independent role of *HIPK2:* rs2030712 genotypes in RP.

## Discussion

In this study, *HIPK2:* rs2030712 were found for the first time to be significantly associated with the occurrence of RP ≥ grade 2. Patients with the CC genotype of *HIPK2:* rs2030712 had a significantly increased risk of RP after radiotherapy for lung cancer. We also discovered that the association between *HIPK2:* rs2030712 and RP grade ≥ 2 was independent of MLD and V_20_.

The occurrences of RP ≥ grade 2 were 58.6%, which were similar to those reported previously. Due to the prospective nature of our study, the incidence rate of RP was relatively higher than in some retrospective studies. We also confirmed that age, MLD and V_20_ was closely related to the risk of RP. In our cohort, patients with MLD ≥ 15Gy and V_20_ ≥ 24% had a greater risk of developing RP grade ≥ 2, which verified the associations between the radiation dosimetric-related factors and the occurrence of RP.

As is well known that pro-inflammatory and fibrogenic cytokines induced by irradiation are involved in the pathogenesis of RP [21]. Our previous studies have already demonstrated that SNPs of several genes involving inflammation regulation are associated with RP risk [11–14]. However, the exact role that *HIPK2* playing in tissue inflammation and fibrosis is largely unknown. Recent advances in kidney fibrotic disease indicated that the protein expression of *HIPK2* was significantly elevated in human HIV- associated nephropathy patients [22]. In renal tubular epithelial cell model, study demonstrated that *HIPK2* not only up-regulated expression of several pro-fibrotic cytokines, such as smooth muscle actin, fibronectin, collagen I, but also activated several pro-fibrotic and pro-inflammatory signal pathways including TGF- β (transforming growth factor β)–Smad3, Wnt-Notch and NF-κB pathways [23]. On the other hand, the role of *HIPK2* in pulmonary fibrotic disease, especially in RP is much less clear. Results from study on idiopathic pulmonary fibrosis (IPF) patients demonstrated that *HIPK2* expression in IPF-derived fibroblasts is significantly lower compared with normal counterparts [19]. In addition, allelic deletion was detected specifically in IPF fibroblast, which may be caused by chronic inflammation. This phenomenon was shown to be similar with precancerous lesion, which defective *HIPK2* was accumulated during clonal expansion of IPF fibroblast under inflammatory stimulus. Therefore, *HIPK2* may represent a novel therapeutic target in pulmonary fibrotic diseases.

Furthermore, in this study we demonstrated for the first time the prevalence and clinical value of *HIPK2:* rs2030712 on RP in independent Chinese Han cohort, and may thus be one of the important predictors of severe RP before radiotherapy in addition to the radiation dosimetric factors. Those patients with RP susceptibility genotypes will greatly benefit from early prediction and prevention of RP by genotyping before the initiation of RT. And this study will help us to choose the patients without RP susceptibility genotypes and elevate their radiation dose appropriately for a better control of tumor. Especially for the patients with favorable genotypes, elevated MLD and V_20_ will not increase their incidence of severe RP, which could assist the oncologist to adjust the radiation dose personally. Moreover, our findings suggest the possible role of *HIPK2* in the pathogenesis of RP, which will aid in the discovery of target to treat RP in future research.

On the other hand, interstitial lung disease (ILD) is one of the risk factors that have been demonstrated to be related with increased RP incidence [24]. Unfortunately, in this study we didn’t include the status of ILD as clinical parameter for cohort analysis, which had potential influence in our conclusion. Therefore, our results still require further validation in expanded cohorts with related ILD status information from different races, since the substantial ethnic variation exist in SNP frequencies. Moreover, *HIPK2:* rs2030712 warrant further investigation to identify the causative SNPs and their molecular mechanisms. Furthermore, we need to explore the potential role of *HIPK2* pathway in the pathogenesis of RP, which would provide novel insight into the treatment of RP.

## Conclusions

In summary, it is the first study to confirm the associations between RP risk and *HIPK2:* rs2030712, and thus indicated that in addition to the radiation dosimetric factors, *HIPK2* SNP can be used as useful predictive biomarker of RP risk before RT. Thus, patients will greatly benefit from early prediction and prevention of RP by genotyping before the initiation of RT. And this study will benefit lung cancer patients receiving radiotherapy since appropriately tailored radiation dose might result in better control of their diseases and lower occurrence and severity of RP.

## Data Availability

The detailed genetic data from our cohort analyzed during the current study are not publicly available due to the private gene information protection policy of our center. But they are available from the corresponding author on reasonable request.

## Abbreviations

IPF: Idiopathic pulmonary fibrosis
KPS: Karnofsky performance status
MLD: Mean lung dosage
NSCLC: Non small cell lung cancer
RP: Radiation pneumonitis
RT: Radiotherapy
SCLC: Small cell lung cancer
SNP: Single nucleotide polymorphism
